# Robust error calibration for serial crystallography

**DOI:** 10.1107/S2059798325002852

**Published:** 2025-04-29

**Authors:** David W. Mittan-Moreau, Vanessa Oklejas, Daniel W. Paley, Asmit Bhowmick, Romie C. Nguyen, Aimin Liu, Jan Kern, Nicholas K. Sauter, Aaron S. Brewster

**Affiliations:** a Molecular Biophysics and Integrated Bioimaging Division Lawrence Berkeley National Laboratory Berkeley CA94720 USA; b Department of Chemistry, University of Texas at San Antonio, San Antonio, TX78249, USA; University of Cambridge, United Kingdom

**Keywords:** uncertainty quantification, error calibration, serial crystallography, XFELs

## Abstract

Serial crystallography is an important technique with unique abilities to resolve enzymatic transition states, minimize radiation damage to sensitive metallo­enzymes and perform *de novo* structure determination from micrometre-sized crystals. This technique requires the merging of data from thousands of crystals, making manual identification of errant crystals unfeasible. *cctbx.xfel.merge* uses filtering to remove problematic data. However, this process is imperfect, and data reduction must be robust to outliers. We add robustness to *cctbx.xfel.merge* at the step of uncertainty determination for reflection intensities. This step is a critical point for robustness because it is the first step where the data sets are considered as a whole, as opposed to individual lattices. Robustness is conferred by reformulating the error-calibration procedure to have fewer and less stringent statistical assumptions and incorporating the ability to down-weight low-quality lattices. We then apply this method to five macromolecular XFEL data sets and observe the improvements to each. The appropriateness of the intensity uncertainties is demonstrated through internal consistency. This is performed through theoretical CC_1/2_ and *I*/σ relationships and by weighted second moments, which use Wilson’s prior to connect intensity uncertainties with their expected distribution. This work presents new mathematical tools to analyze intensity statistics and demonstrates their effectiveness through the often underappreciated process of uncertainty analysis.

## Abstract 2

We present a new error-calibration algorithm for reflection intensities in serial crystallography. We reformulate the mathematical basis of the problem and apply different levels of uncertainty to each observed lattice corresponding to its measurement accuracy. These uncertainties are more consistent with theoretical expectations than the Ev11 error-calibration algorithm previously implemented in *cctbx.xfel.merge*.

## 1. Introduction

In macromolecular crystallography (MX), data reduction is the conversion of raw frames of X-ray diffraction into averaged structure-factor intensities and uncertainties for subsequent structural modeling. Diffraction patterns are first indexed to determine the location of each reflection. The number of photons scattered into each reflection is then summed to form a list of integrated intensities, with uncertainties derived from counting-statistics errors. These values are scaled to place them onto a common magnitude and to correct for known effects that modulate intensities. Counting-statistics error thus forms the lower bound for uncertainty, because it does not account for errors in the scaling process or other experimental variances. Many of these sources of error have been enumerated in the literature (Holton *et al.*, 2014 ▸; Diederichs, 2010 ▸). Assuming that each error source can be modeled explicitly, a textbook approach would be to form the final uncertainty estimate by propagating each contribution explicitly. In the cases of small errors and/or linear models, first-derivative approaches are used. Otherwise, sampling techniques such as Markov chain Monte Carlo algorithms or variational inference are used (Possolo & Iyer, 2017 ▸; Bevington & Robinson, 2003 ▸).

In practice, however, error sources are incompletely known, so crystallographic data reduction has historically taken an empirical approach towards error modeling (Busing & Levy, 1957 ▸; McCandlish *et al.*, 1975 ▸). Sources of a reflection’s error, beyond counting-statistics error, are accounted for by an empirical transformation of the scaled counting-statistics error (Leslie, 1999 ▸; Borek *et al.*, 2003 ▸; Evans, 2006 ▸, 2011 ▸; Kabsch, 2010*a* ▸; Diederichs, 2010 ▸; McCandlish *et al.*, 1975 ▸; Evans & Murshudov, 2013 ▸; Brewster, Bhowmick *et al.*, 2019 ▸; Beilsten-Edmands *et al.*, 2020 ▸; Khouchen *et al.*, 2023 ▸). This approach to uncertainty quantification is known as error calibration and shares many similarities with statistical post-processing in weather forecasting (Vannitsem *et al.*, 2021 ▸), astronomy (Chen *et al.*, 2019 ▸) and machine learning (Palmer *et al.*, 2022 ▸; Kuleshov *et al.*, 2018 ▸; Levi *et al.*, 2022 ▸). This approach is justified by the needs of MX data-reduction programs and existing characterization of experimental X-ray sources. These programs operate in an automated manner on data from a wide variety of samples collected from sources with limited, and often inaccurate, characterization (Winter, 2010 ▸). The lack of knowledge of the root sources of uncertainty, such as unit-cell distribution, point-spread functions, parallax and detector response, prevents our ability to explain the error we see in the observed intensities, necessitating this empirical approach.

Most data-reduction programs use the transformed errors as weights to average redundant measurements (Kabsch, 2010*b* ▸; Otwinowski *et al.*, 2012 ▸; Beilsten-Edmands *et al.*, 2020 ▸; Evans, 2006 ▸). This approach is taken in *cctbx.xfel.merge* and *xia*2.*ssx*. An alternative approach to merging is to average the scaled measurements together without weighting and use the residuals as the final uncertainty. This approach is similar to that of Chapman *et al.* (2011 ▸) and *CrystFEL* (White *et al.*, 2012 ▸). Averaging with inverse variance weighting is the maximum-likelihood estimate of the mean. This should improve mean estimates by giving reflections deemed to be more accurately measured by the uncertainty estimate a larger contribution to the average. Brewster, Bhowmick *et al.* (2019 ▸) demonstrated that weighted averaging improved merging results in serial crystallography (SX) only when the transformed errors are used as weights. Weighted averaging using counting-statistics error was worse than unweighted averaging.

This paper details the adaptation of the *cctbx.xfel.merge* Ev11 error model to better reflect SX data and experimentation. *cctbx.xfel.merge* is a program for scaling and merging SX data and is part of the *cctbx.xfel* suite (Brewster, Young *et al.*, 2019 ▸).^1^ The Ev11 error model is the direct implementation of the error model of Evans (2011 ▸) for single-crystal rotational diffraction to SX (Brewster, Bhowmick *et al.*, 2019 ▸; Brewster *et al.*, 2018 ▸). However, SX data, experimentation and interpretation differ in ways that justify an SX-specific error model (Gorel *et al.*, 2021 ▸). In rotational crystallography, a single crystal is rotated while being illuminated by a stable, ‘continuous’ beam. This rotation allows the integration of the full three-dimensional profile of the diffracted intensity and ensures smooth continuity between frames. An assumption can be made that crystal properties and quality remain relatively constant for the entire data set while scaling factors slowly vary. In SX, data sets are built up by separately collecting data from thousands of randomly oriented crystals, without rotation, and with an incident beam varying in both intensity and wavelength. A SX error model must be robust to outlier data and capable of applying different degrees of error to each lattice according to its accuracy in measurement and scaling.

For the new empirical error model, we are attempting to find a transformation of the initial counting-statistics uncertainties that explains the scale of the distribution of redundant measurements. We apply concepts of robust statistics (Lange *et al.*, 1989 ▸) to the determination of this model. Robustness describes the ability of an algorithm to resist failure when subjected to data that violate the statistical assumptions made on the data. Robustness can be increased by using fewer and less stringent assumptions. Our robust approach requires the writing of a likelihood distribution. Unfortunately, SX data do not follow simple, easily derived distributions (Sharma *et al.*, 2017 ▸). Therefore, a likelihood function is written based on pairwise differences of symmetry-related reflections. Generally speaking, pairwise differences of samples drawn from the same distribution can be used as a measure of the scale of the distribution irrespective of its mean or skew (Rousseeuw & Croux, 1993 ▸). This represents a reduction in assumptions. To model our data, we write the likelihood function utilizing a *t*-distribution. This distribution has longer tails than a normal distribution and is more accommodative to outlier data, forming a less stringent assumption.

In serial crystallography, diffraction frames are recorded. These frames can comprise diffraction from multiple crystals. A lattice refers to the diffraction from a single crystal. Differing degrees of error are applied to each lattice based on the Pearson correlation coefficient between the reflection intensities of a lattice and a scaling reference. This scaling reference can be either a PDB file or, in the case of *de novo* structure determination, a data set merged without a scaling reference. These correlations form a continuous variable that, in principle, should have a monotonic relationship with measurement, scaling and correction accuracy. The Ev11 parameterized error transformation is rewritten so different levels of error are applied to each lattice based on this correlation coefficient.

The application of robust statistics to MX includes peak finding (Hadian-Jazi *et al.*, 2017 ▸), the generation of bad pixel masks (Sadri *et al.*, 2022 ▸), background modeling (Parkhurst *et al.*, 2016 ▸) and finding pseuodotranslations (Sauter & Zwart, 2009 ▸). Recent efforts have applied robust statistics to scaling and merging (Aldama *et al.*, 2023 ▸; Greisman *et al.*, 2021 ▸; Dalton *et al.*, 2022 ▸). Greisman *et al.* (2021 ▸) describe a maximum-likelihood approach to merging data with an error modeling that utilizes a *t*-distribution for robustness. Distributions for the merged intensities are inferred from the distribution of the redundant measurements. Their further work on this subject (Dalton *et al.*, 2022 ▸) uses deep-learning-based variational inference to infer distributions of merged structure factors. Their methods utilize a *t*-distribution to model reflection intensities robustly. Our error model differs by determining uncertainties for unmerged reflections so that reflections assigned high uncertainty can be down-weighted during merging.

The uncertainty estimates of intensities are first used in the merging process, the weighted average of symmetry-related and multiply measured observations. Improved merging produces more accurate intensities, which should generate higher quality electron-density maps. Uncertainties are also relevant throughout structure determination and refinement. *Phenix* utilizes reflection uncertainties for outlier rejection (Read, 1999 ▸), pruning low-information reflections (Read *et al.*, 2020 ▸), French–Wilson conversion (French & Wilson, 1978 ▸), phasing and molecular replacement (Read & McCoy, 2016 ▸; Brewster, Bhowmick *et al.*, 2019 ▸), and structure refinement (Lunin *et al.*, 2002 ▸).

We demonstrate improved uncertainty estimates through their consistency with other statistical quantities. The direct impact of this on merging is shown by improvements to merging statistics. The more distant impact on electron-density maps is shown through increased anomalous map heights at heavy-atom positions.

## 2. Methods

### 2.1. Overview of the previous Ev11 error model

We now rederive the Ev11 method presented by Brewster, Bhowmick *et al.* (2019 ▸) to examine its assumptions and identify avenues of improvement that will be presented in the next section. The following notation will be used. If *R* is normally distributed with mean μ and variance σ^2^, this will be denoted as 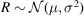. The expected value and expected standard deviation of *R* are *E*(*R*) and std(*R*), respectively.

Before error calibration, a post-refinement process is performed that scales intensities and counting-statistics uncertainty to place them on the scale of a common reference. This corrects for lattice-to-lattice intensity variations due to incident beam intensity, illuminated crystal volume and Wilson *B* factor, and includes per-observation corrections for partiality. These factors are discussed in Brewster, Bhowmick *et al.* (2019 ▸) and Sauter (2015 ▸).

The Ev11 error model, detailed in Brewster, Bhowmick *et al.* (2019 ▸) and Evans (2011 ▸), approaches the problem of error calibration by utilizing the large redundancy in an XFEL crystallographic data set. Ignorance of the underlying sources of error is assumed. A parametric form of uncertainty is written for each reflection and is optimized to explain the observed variance within the data.

The intensity of the *k*th observation of Miller index *h* is written as *I*_*hk*_ and is assumed to be normally distributed with mean *I*_*h*_, the true unobserved intensity, and variance 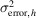,
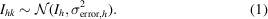
It is assumed that each observation of Miller index *h* is independently and identically distributed and that all symmetry-related reflections have been grouped into a common Miller index. The distribution of observations of the intensity of a Bragg peak has been suggested to not be normally distributed (Sharma *et al.*, 2017 ▸) and it is not generally true that each observation of Miller index *h* is identically distributed. Our investigation of intensities after post-refinement also suggests a complex, non-normal distribution, an observation that we will return to later.

The Ev11 algorithm starts by writing a parameterized form of the measurement error, 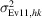, that can be optimized to approximate the true, unknown error, 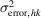,
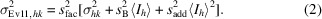
In this equation, 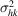 is the counting-statistics uncertainty derived from spot integration and 〈*I*_*h*_〉 is the average intensity of all measurements of reflection *h*. The terms *s*_fac_, *s*_B_ and *s*_add_ are optimizable parameters introduced by Evans (2011 ▸). The *s*_fac_ parameter scales the intensities and counting-statistics uncertainty to account for error in the detector gain. The *s*_add_ parameter accounts for undescribed sources of measurement error that should have variances that scale with 〈*I*_*h*_〉^2^. The *s*_B_ parameter is given no physical meaning. Evans (2011 ▸) included this term because it seemed to improve the fitting to experimental data. These parameters are global; they have the same value for each lattice.

The Ev11 algorithm optimizes *s*_fac_, *s*_B_ and *s*_add_ such that 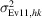 explains the observed residual or difference between *I*_*hk*_ and 〈*I*_*h*_〉. Normalized deviations are introduced as part of this optimization as a ratio of the observed residual to 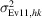. When the Ev11 error terms are correctly optimized, the distribution of these normalized deviations should have a standard deviation of one, which we will take advantage of to build a target function. The derivation of the normalized deviations starts with 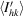, the mean of the *n* observations of Miller index *h*, except for the *k*th reflection. We can then write 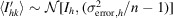. The *n* − 1 term comes from averaging *n* − 1 reflections under the assumption that each observation of Miller index *h* is independently and identically distributed. The residuals are distributed as 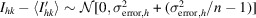, where the variances add linearly. The normalized deviations for each measurement are constructed by dividing 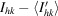 by our approximation of the measurement error, 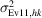,
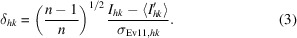
When 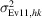 is an accurate estimate of 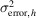, the standard deviation of all δ_*hk*_ for a common Miller index *h* should be one and can be estimated as the root-mean-squared δ_*hk*_, 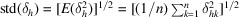, given that the expected value of the normalized deviations, *E*(δ_*h*_), is zero. Ev11 therefore refines *s*_fac_, *s*_B_ and *s*_add_ to minimize the difference in the standard deviation of δ_*hk*_ from one.

The creation of the target function starts by binning the reflections based on the mean observed intensity of all of their symmetry-related reflections 〈*I*_*h*_〉. 100 intensity bins are created, evenly spaced in intensity ranging from the minimum to maximum 〈*I*_*h*_〉. All reflections of a Miller index are assigned to a single bin, indexed by *b*, based on their associated 〈*I*_*h*_〉. A subscript *b* is introduced to indicate the binning and the Miller indices within a bin are denoted by *h*_*b*_.

The target function used to optimize *s*_fac_, *s*_B_ and *s*_add_ minimizes the difference between our estimate of 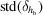 within an intensity bin and one,
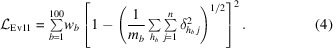
The weighting for each bin is the square root of the number of observations in the bin *b*, *w*_*b*_ = (*m*_*b*_)^1/2^. If the weighting was chosen to be one for all bins, it would weight each intensity bin equally. On the other hand, if we choose *w*_*b*_ = *m*_*b*_, it would weight each observation equally. The choice of *w*_*b*_ = (*m*_*b*_)^1/2^ is a middle ground between these two options. Minimization is performed using the *scitbx* L-BFGS-B optimizer (Zhu *et al.*, 1997 ▸) with analytical first derivatives.

### 2.2. MM24: updated error model

A new error model is developed using the Ev11 approach with several key differences. (i) The *s*_B_ parameter is removed and *s*_add_ is further parameterized to reflect different uncertainty levels between frames. (ii) Pairwise differences of symmetry-related reflection intensities are used as a robust measure of their standard deviation, as opposed to the normalized deviations in the Ev11 protocol. (iii) The optimization target function is replaced with a maximum log-likelihood target function. (iv) Intensity binning is removed from the loss function. (v) A new initialization algorithm is used.

The Ev11 protocol uses a constant *s*_add_ for each lattice. To account for the lattice-to-lattice differences in the measurement accuracy, *s*_add_ is written as an exponential decay of the Pearson correlation coefficient, cc_*l*_, of the measured reflections of lattice *l* to a supplied scaling reference,
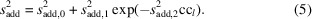
In this equation, there are three optimized coefficients *s*_add,α_. When *s*_add,1_ = 0, *s*_add_ is a constant for all frames. For nonzero *s*_add,2_, 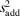 increases as *cc*_*l*_ decreases. Each *s*_add,α_ coefficient is the same for all lattices. *s*_add_ is lattice-specific because cc_*l*_ differs between lattices. The exponential form was chosen because it produces a curve that matches expectation: 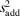 should always be positive and decrease monotonically with increasing cc_*l*_. It also restrains the curve to prevent numerical issues. For example, if a polynomial was used instead, it could result in a form that crosses zero, resulting in a division-by-zero error. The 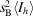 term is removed from equation (2), as also performed by Beilsten-Edmands *et al.* (2020 ▸). This term has some redundancy because 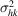 and 〈*I*_*h*_〉 are correlated. In practice, *s*_B_ tends to optimize to relatively small values and the 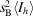 term has a trivial contribution. We do not observe the 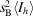 term improving fits to experimental data in the same way that Evans (2011 ▸) did to justify its initial inclusion. These changes give a new uncertainty equation
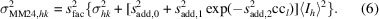
The derivation of the normalized deviations in Ev11 assumed that they were identically distributed: symmetry-related peaks had the same variance, required an estimate of mean 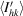
and assumed that the normalized deviations are distributed symmetrically about zero. A target function can be derived based on pairwise differences of symmetry-related reflections without making these assumptions. Pairwise differences can be used to robustly quantify the variance of a distribution without an estimate of a mean (Rousseeuw & Croux, 1993 ▸) and are guaranteed to be an even function, symmetric about zero. A set of normalized pairwise differences is constructed as
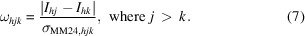


Here, 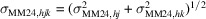 represents the uncertainty of the pairwise difference. This is assembled given that uncertainties add in quadrature for normally distributed random variables.

If we assume that 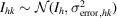, which does not assume that all measurements of Miller index *h* have the same variance, unlike equation (1) for the normalized deviations, then 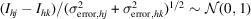. The normalized pairwise differences are then distributed as a half-normal variable and the likelihood function is
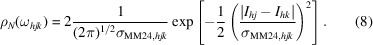
This likelihood could be replaced with a *t*-distribution to account for data distributed with longer tails than a normal distribution or to make the optimization more robust to outliers. We will use likelihood to optimize equation (6), but maximum-likelihood optimization with a normal distribution is sensitive to outliers. To increase the optimization robustness, we choose to approximate this distribution as a half *t*-distribution,
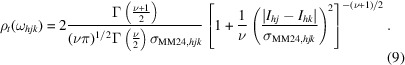
The gamma function, Γ(*x*), is equivalent to (*x* − 1)!; however, it is defined for non-integer numbers. The parameter ν is known as the degrees of freedom. The *t*-distribution models the difference between the true mean of a random variable and the mean estimated from *n* samples. In this interpretation, ν = *n* − 1. In our implementation, where the *t*-distribution is used as a generic probability distribution, ν can be viewed as a tuning parameter that adjusts the shape of the distribution. When ν = 1, the *t*-distribution is equivalent to the long-tailed Cauchy distribution. As ν approaches infinity, the *t*-distribution converges to the normal distribution.

A loss function is written that minimizes the negative of the log-likelihood of the pairwise differences,
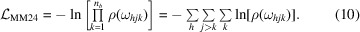
The binning scheme from the Ev11 protocol is removed from the loss function; however, it is used later for a new initialization procedure.

For the thermolysin data set utilized in this study, there are of the order of 10^7^ unmerged reflections. The number of pairwise differences makes the optimization based on all possible pairwise differences intractable due to limited computational memory. To reduce memory demands, a maximum of 100 pairwise differences are randomly sub­sampled for each Miller index and used for optimization. To ensure reproducibility for distributed computing, a new random-number generator is created for each set of reflections with a seed calculated from the common Miller indices. Cantor’s pairing function maps two natural numbers to one unique natural number, π(κ_1_, κ_2_) = [(κ_1_ + κ_2_)(κ_1_ + κ_2_ + 1)]/2 + κ_2_), and is used to convert Miller indices to a unique seed. Each Miller index is increased by 1000, to ensure they are all positive, and Cantor’s pairing function is then iteratively applied. An optional integer can be supplied that is added to the unique seed to obtain different results. The parameters *s*_fac_, *s*_add,0_, *s*_add,1_, *s*_add,2_ and, when using a *t*-distribution, ν, are optimized by minimizing equation (10) using the *scitbx*L-BFGS-B optimizer (Zhu *et al.*, 1997 ▸) with analytical first derivatives.

In Ev11, *s*_fac_ and *s*_add_ are initialized using the normalized deviations. Because they are not calculated in MM24, a new algorithm is used to initialize *s*_fac_ and *s*_add,1_. The terms *s*_add,0_ and *s*_add,2_ are initialized to 0.001. A set of unnormalized pairwise differences are constructed, 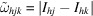. Their means, 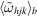, are calculated in 100 intensity bins spanning zero and one tenth of the maximum biased mean, 0.1max(〈*I*_*h*_〉). Zero is chosen as the lower limit for numerical stability because a square root of this value is included. The upper limit of one tenth of the maximum reflects that the bulk of the reflection intensities tends to be at least one order of magnitude less than the maximum. If *I*_*hj*_ and *I*_*hk*_ are sampled from a normal distribution with mean *I*_*h*_ and variance 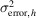, then *I*_*hj*_ − *I*_*hk*_ follows a normal distribution with zero mean and variance 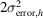 and 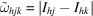 follows a half-normal distribution with variance 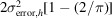. The mean of 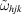 is then 2/π^1/2^σ_error,*h*_. Therefore, 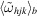 should scale with 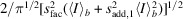. 〈*I*〉_*b*_ is the central intensity of the bins. The *s*_fac_ and *s*_add,1_ terms are initialized by the minimization of
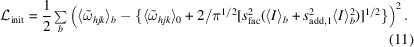
The term 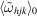 is the mean pairwise difference of the first positive-intensity bin and represents the expected nonzero error of low-intensity reflections.

### 2.3. Weighted second moments

The second moment of intensity, 〈*I*^2^〉/〈*I*〉^2^, is a metric commonly used to identify twinning (Stanley, 1972 ▸). The mean intensity, 〈*I*〉, and mean squared intensity, 〈*I*^2^〉, can be calculated over all merged reflections or in resolution bins. For crystals that are not twinned, the theoretical second moment for acentric reflections is two, as inferred from the Wilson distribution (Wilson, 1949 ▸), in the absence of measurement error. In high-resolution bins, measurement error becomes comparable to the mean intensity. This adds to the dispersion of the intensities and the observed second moment increases from two. The formula for this departure is derived by combining the expected distribution for the intensities within a resolution bin and the measurement error (Read *et al.*, 2020 ▸).

The Wilson distribution tells us how acentric reflection intensities, *I*, should be distributed given the mean intensity, Σ: ρ(*I*) = Σ^−1^exp(−*I*/Σ). Using the normalized intensity, *Z* = *I*/Σ, this simplifies to ρ(*Z*) = exp(−*Z*). If we assume Gaussian measurement error, the distribution of the observed normalized intensity, 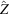, given its true value, *Z*, and estimated measurement error, 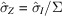, is
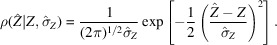
Integrating out the true intensity gives the distribution of the observed normalized intensity given measurement error,
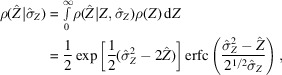
where erfc is the complementary error function. A similar derivation can be found in Read *et al.* (2020 ▸) for centric reflections. This distribution is in the form of an exponentially modified Gaussian distribution with mean 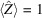 and variance 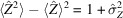. The second moment follows as 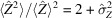. Weighted second moments are calculated in *phenix.phaser* after outlier rejection and anisotropic *B*-factor correction (McCoy *et al.*, 2007 ▸).

## 3. Results

Comparisons of the different error models were made with five data sets: a thermolysin data set (PDB entry 4ow3; Kern *et al.*, 2014 ▸), an unpublished cytochrome data set similar to PDB entry 8tdq, an isopenicillin N synthase (IPNS) data set associated with PDB entry 6zae (Rabe *et al.*, 2021 ▸), a methane monooxygenase hydroxylase (MMOH) data set associated with PDB entry 6yd0 (Srinivas *et al.*, 2020 ▸) and a second methane monooxygenase hydroxylase data set (sMMOox) that is unpublished.

The thermolysin data set is available as cxi.db entry 81 (https://www.cxidb.org/id-81.html). It was collected at a wavelength of 1.27 Å with anomalous scattering from Zn and Ca atoms, which have absorption edges at 1.2837 and 3.0705 Å, respectively. Comparisons with thermolysin were made using phasing results in the same manner as the previous error-model and merging comparisons (Brewster, Bhowmick *et al.*, 2019 ▸; Dalton *et al.*, 2022 ▸). Integration results were taken directly from the earlier work (Brewster, Bhowmick *et al.*, 2019 ▸). Merging used PDB entry 4ow3 as the scaling reference and a correlation outlier filter of 0.1. This outlier filter works by removing lattices whose correlation between the observed intensities and a scaling reference is less than a set threshold. This correlation is determined before post-refinement is applied. Phasing and autobuilding are performed using *phenix.autosol* with an exact amino-acid sequence and two Zn atoms specified. 30 trials with different random seeds were performed. The anomalous map height of one Zn atom is calculated using *xfel.map_height_at_atom* with the merged reflections and each *phenix.autosol* solution. The number of residues built, *R*_work_ and *R*_free_ metrics were determined using the structures produced from *phenix.autosol.* Table 1 ▸ reports the mean and standard deviation of the trials. The left column of Table 1 ▸ shows the results using the Ev11 protocol, as performed in Brewster *et al.* (2018 ▸) and the right column shows the MM24 protocol with a *t*-distribution likelihood.

For the cytochrome, IPNS, MMOH and sMMOox data sets, the images were first indexed and integrated with *dials.stills_process* (Brewster *et al.*, 2018 ▸). The integration results were merged using *cctbx.xfel.merge* with PDB entries 7s0o, 6zae, 6yd0 and 6ydi as scaling references. A correlation outlier filter of 0.1 was applied to IPNS and MMOH and 0.3 for cytochrome to be consistent with the published work. It was not applied to sMMOox. Our standard practice is to not use an outlier filter as it removes lattices. When there is reason to suspect that low-quality data are being measured, we switch to a correlation filter of 0.1. During live data collection of sMMOox, the correlation filter was not used. At the time of publication, PDB entry 8tdq superseded PDB entry 7s0o for cytochrome. Cytochrome contains a heme-bound Fe atom, IPNS contains a Fe atom and MMOH and sMMOox contain two Fe atoms. The anomalous map heights at these atoms are calculated using *xfel.map_height_at_atom* using the merged intensity and refined structures. The left column of Table 1 ▸ shows the results using the current Ev11 protocol, including the correlation outlier filter in *cctbx.xfel.merge*. Each set of merged intensities was used as a starting point for 30 different refinements in *phenix.refine* starting from the scaling reference structure. The map heights, *R*_work_ and *R*_free_ values reflect the mean and standard deviation over these trials. The Wilson *B* factor was calculated using *phenix.xtriage*. Data and detailed processing details are available for cytochrome in cxi.db entry 229 (https://www.cxidb.org/id-229.html) and those for IPNS and MMOH in cxi.db entry 230 (https://www.cxidb.org/id-230.html).

For the results in Table 1 ▸, for cytochrome, IPNS, MMOH and sMMOox high-resolution cutoffs were made at the point where the multiplicity in the highest resolution shell was at ten and the resolution-binned CC_1/2_ was declining monotonically. This is the standard rule of thumb that we apply to SX data processing and we have found that it gives consistent and reliable results. Thermolysin data were cut at 1.8 Å resolution to be consistent with Brewster, Bhowmick *et al.* (2019 ▸). Including data beyond this point reduced anomalous peak heights. The total number of lattices, observed reflections and unique reflections are listed in Table 1 ▸ and are the same for the MM24 and Ev11 protocols. For the MM24 protocol, the degrees of freedom of the *t*-distribution are optimizable parameters and their optimized values are listed in Table 1 ▸. Generally, several trends are observed for the MM24 protocol, *I*/σ is larger and CC_1/2_ increases. The map heights show a small but significant increase at the heavy-atom sites. Cytochrome shows a large increase in map height and improvements in the refined *R*_work_ and *R*_free_ metrics.

**Figure 1:**
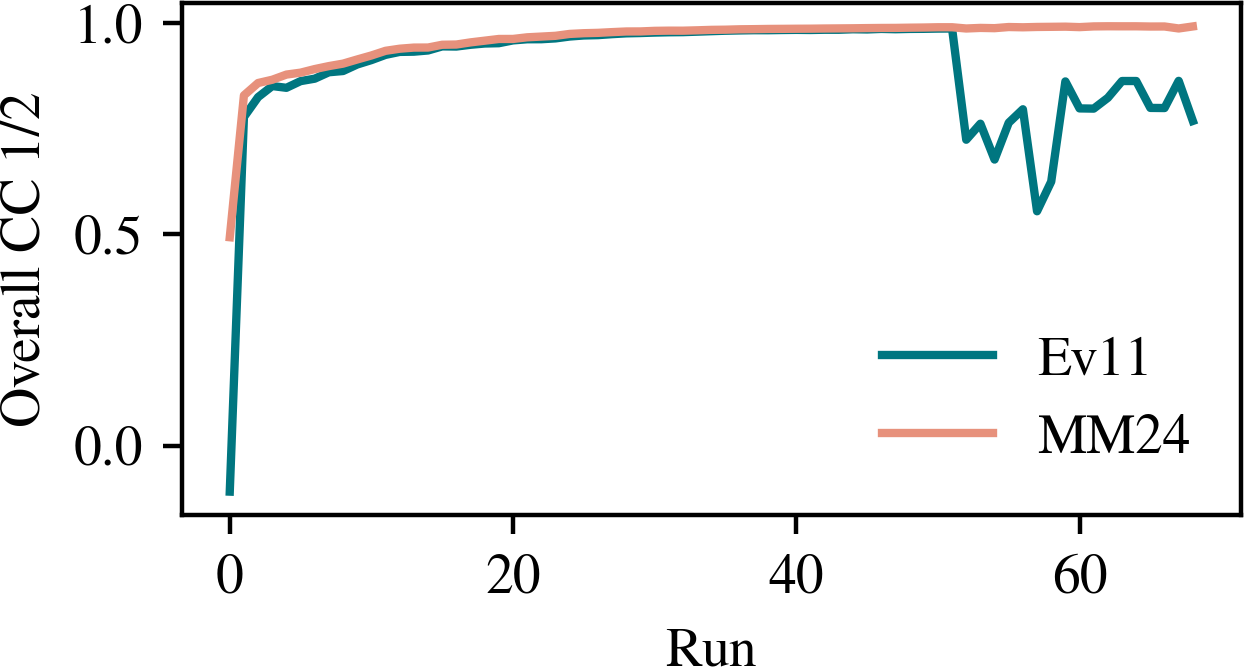
Cumulative CC_1/2_ versus run for sMMOox. During the 52nd run of data collection, a single errant lattice caused the decrease in CC_1/2_ when processed with Ev11. This was not observed with the MM24 algorithm.

During an SX experiment, processing data in real time is critical for decision making regarding experimental logistics; for example, determining when data collection for a sample can be stopped. The authors use CC_1/2_ as a critical metric for these decisions. For sMMOox, an errant frame was processed during the 52nd run. Fig. 1 ▸ shows that this caused a significant decrease in CC_1/2_ when processed with Ev11. This was not observed with the MM24 algorithm due to the increased robustness of its statistical approach.

**Figure 2:**
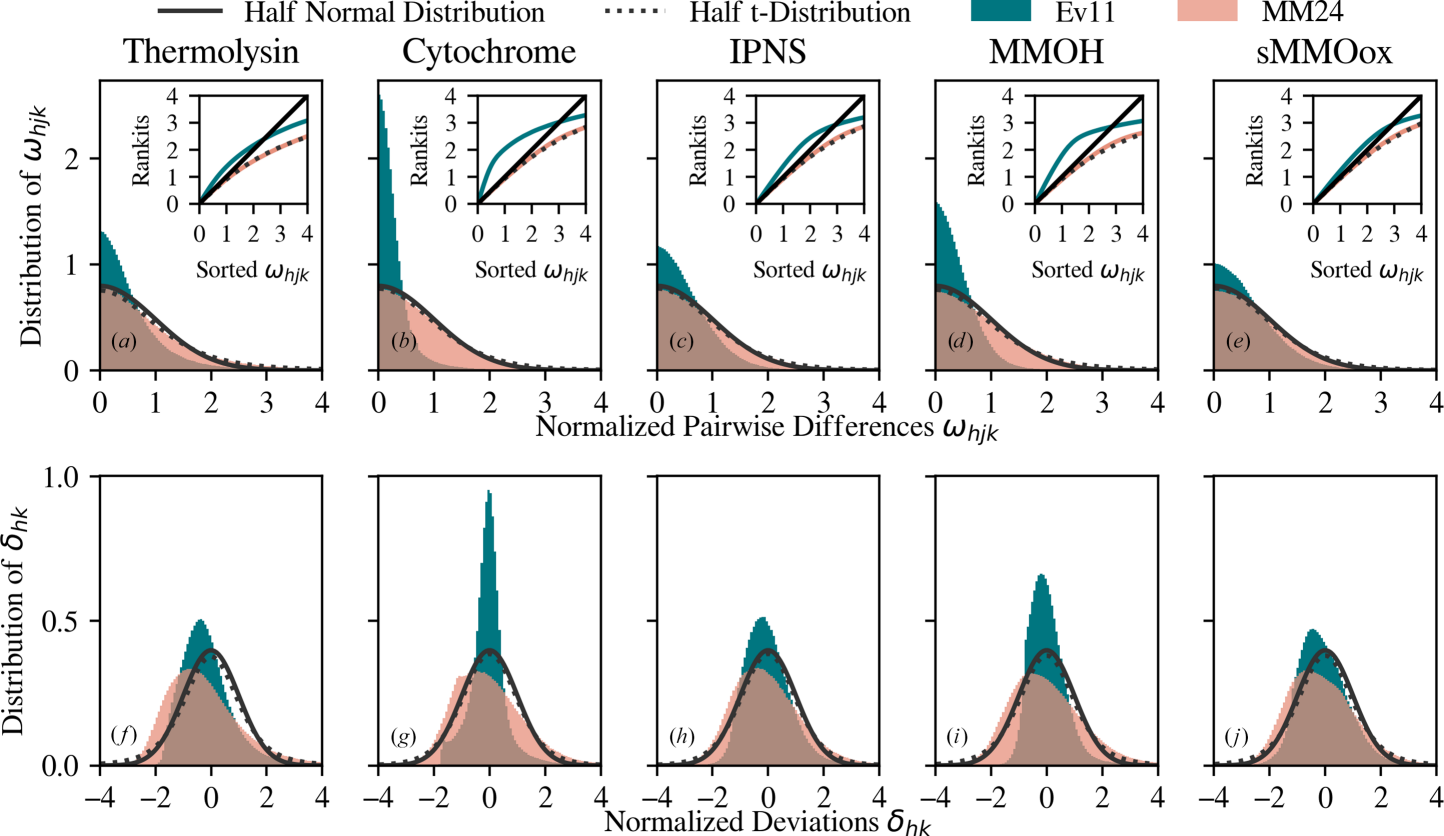
Comparisons of optimization metrics with their target values. The top row shows statistical analysis of the normalized pairwise differences, ω_*hjk*_, for (*a*) thermolysin, (*b*) cytochrome, (*c*) IPNS, (*d*) MMOH and (*e*) sMMOox. The bottom row, (*f*)–(*j*), shows corresponding plots made with the normalized deviations, δ_*hk*_. The MM24 protocol uses a *t*-distribution likelihood with degrees of freedom optimized. In each plot, a histogram of the statistical metrics is plotted for the Ev11 (blue) and MM24 (pink) protocols along with a standard normal distribution (solid black line) and *t*-distribution (dotted black line). The inset in the top row shows the normal probability plots. The solid and dotted lines show the expected curve if the data follow a normal or *t*-distribution, respectively. Deviations from these lines indicate deviations from the assumed distribution.

Fig. 2 ▸ shows statistical analysis of the normalized pairwise differences ω_*hjk*_ and the normalized deviations δ_*hk*_. Histograms of ω_*hjk*_ and δ_*hk*_ are shown with the Ev11 (blue) and MM24 (pink) error models. The MM24 data correspond to the processing results in the right column of Table 1 ▸. For the Ev11 model, ω_*hjk*_ are calculated using the parameterization determined from the Ev11 optimization procedure. These plots demonstrate three points. Firstly, uncertainty estimates from the MM24 protocol are smaller, resulting in broader distributions of ω_*hjk*_ and δ_*hk*_. Secondly, calibrated uncertainties from MM24 match their target *t*-distribution. To demonstrate this, a standard half-normal distribution is drawn as a solid line and a half *t*-distribution as a dotted line, which match the distribution of ω_*hjk*_. Thirdly, there are difficulties in using δ_*hk*_ as an optimization metric. δ_*hk*_ are shown in the lower row. In each of these plots, the histograms of δ_*hk*_ are skewed and offset from zero. The derivation of the Ev11 target function assumes the mean δ_*hk*_ is zero, which is not true given the observed skew and offset.

Abrahams & Keve (1971 ▸) introduced the usage of normal probability plots to assess the error placed on structure factors, an approach that was also followed in Evans (2011 ▸) and Brewster, Bhowmick *et al.* (2019 ▸). These plots are used to visualize the distribution of the data against a theoretical distribution and are especially useful in accessing the correspondence in the tails. In these plots, the data are first sorted and then the expected value of each point is calculated from its position in the sorted data given the assumption that the data follow a normal distribution. These expected values are called rankits. In the insets of Fig. 2 ▸, the rankits are plotted versus the sorted normalized pairwise differences. If the normalized pairwise differences are distributed according to a half-normal distribution, the plot will be a straight line that passes through the origin with a slope of one, as shown by the solid black line. The corresponding expected line for a half *t*-distribution with the optimized degrees of freedom is shown as a dotted black line, which generally fits the MM24 data.

**Figure 3:**
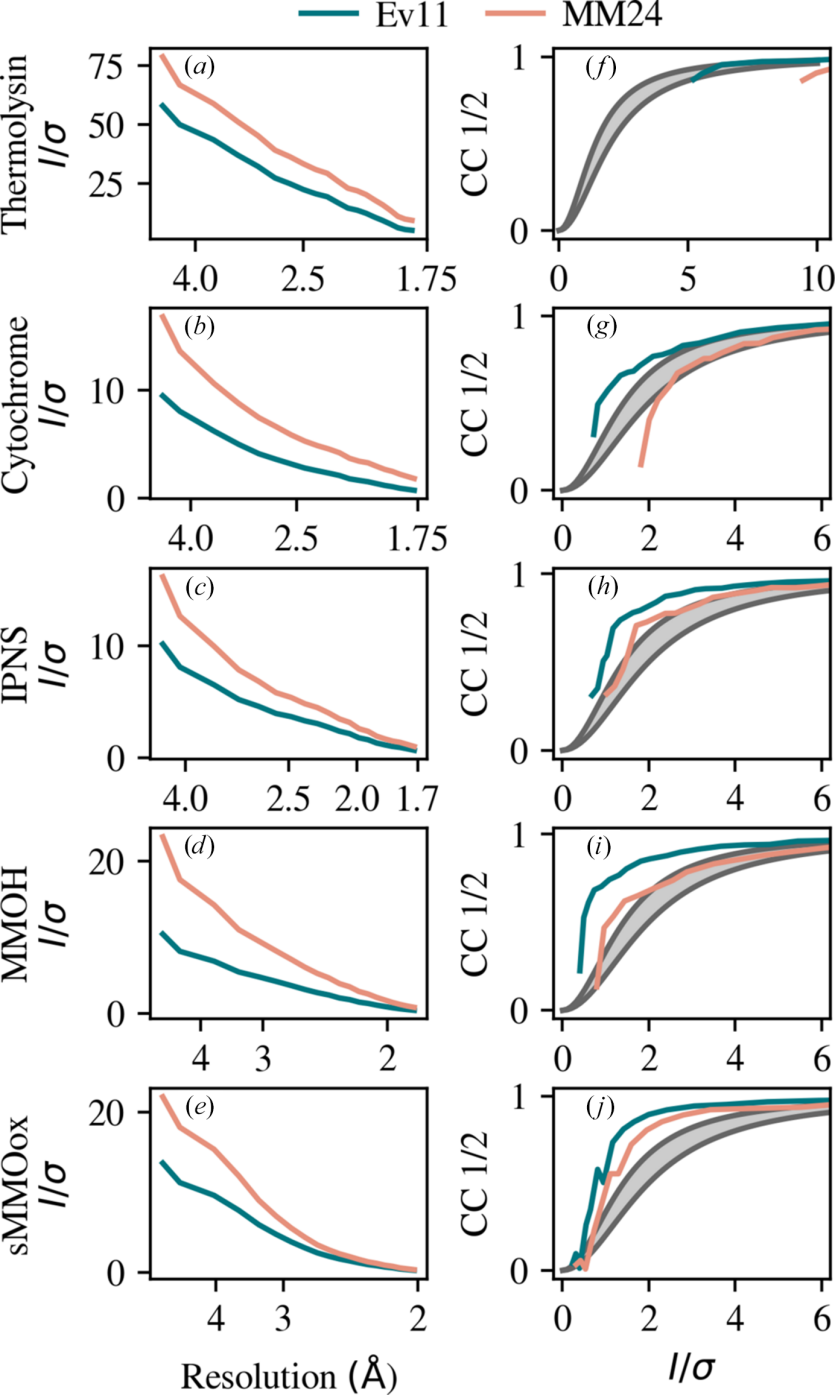
Consistency between CC_1/2_ and *I*/σ. The left column shows the average merged *I*/σ binned by resolution for (*a*) thermolysin, (*b*) cytochrome, (*c*) IPNS, (*d*) MMOH and (*e*) sMMOox. The blue and pink lines correspond to the Ev11 and MM24 protocols, respectively. The right column shows CC_1/2_ plotted against *I*/σ with a gray filled region that corresponds to a theoretical relationship between CC_1/2_ and *I*/σ.

While Fig. 2 ▸ shows that MM24 provides a smaller estimate of uncertainty and the optimization procedure meets its objective, Figs. 3 ▸ and 4 ▸ demonstrate these smaller uncertainties are more consistent with other metrics. Fig. 3 ▸ demonstrates consistency between *I*/σ and CC_1/2_. The left column of Fig. 3 ▸ shows the average merged *I*/σ using Ev11 (blue) and MM24 (pink). The MM24 protocol results in larger *I*/σ throughout the entire resolution range for both data sets due to smaller σ estimates. Because the difference in these algorithms is the estimation of uncertainty, larger *I*/σ values are not indicative of better data. They are simply a result of different uncertainty estimates. To demonstrate internal consistency, the right columns plot CC_1/2_ against *I*/σ with a gray filled region that corresponds to a theoretical relationship between CC_1/2_ and *I*/σ (Karplus & Diederichs, 2015 ▸). Curves lying to the left of the shaded region are due to an underestimation of *I*/σ or, equivalently, an overestimation of σ. Thermolysin was cut at a resolution where CC_1/2_ and *I*/σ are still relatively high. This results in the CC_1/2_ versus *I*/σ curve (Fig. 3 ▸*f*) remaining at a relatively large value.

**Figure 4:**
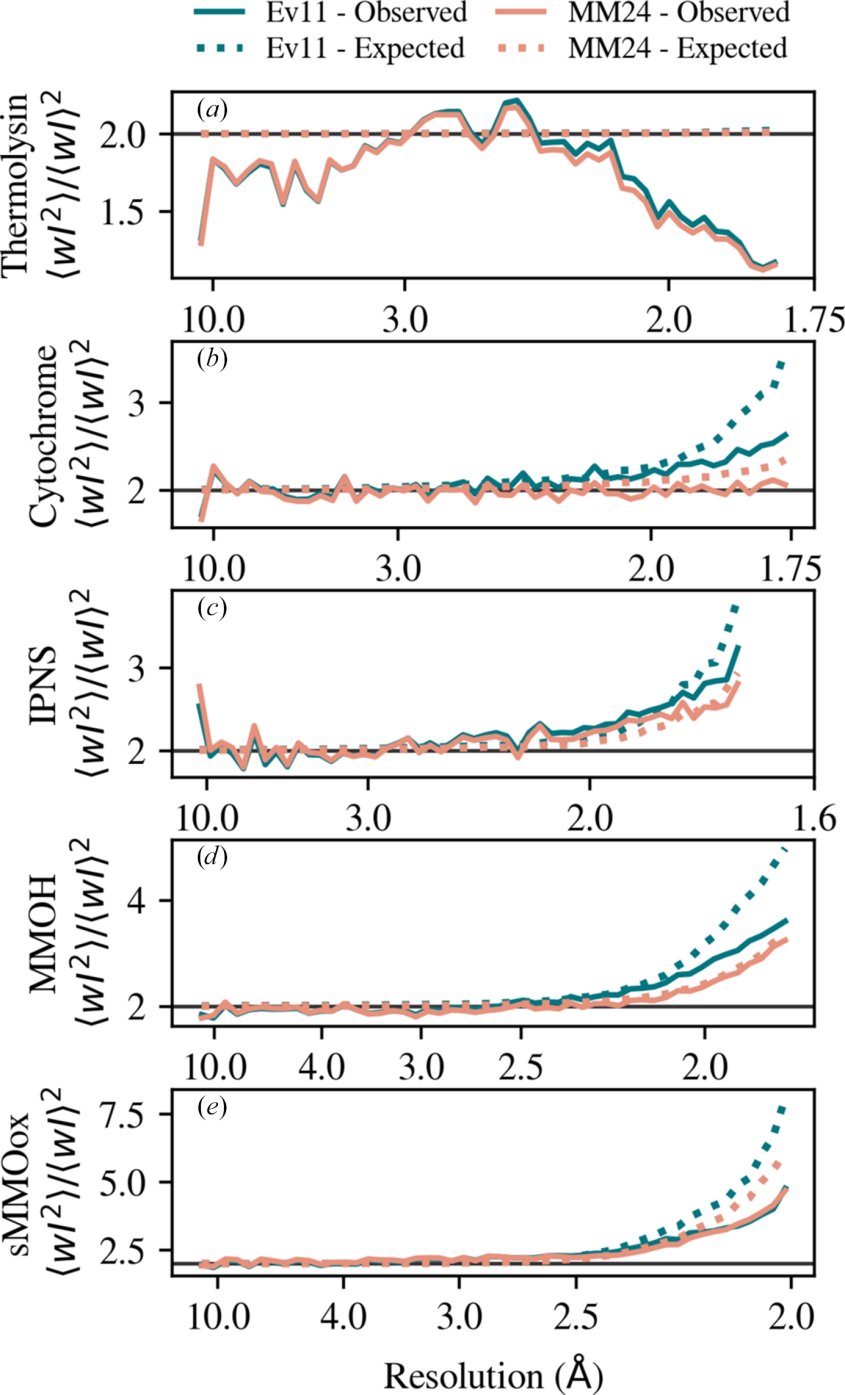
Consistency between *I* and σ. Weighted second moments of the acentric reflections for (*a*) thermolysin, (*b*) cytochrome, (*c*) IPNS, (*d*) MMOH and (*e*) sMMOox as a metric to evaluate the uncertainty estimates of the error model. According to Wilson’s distribution, the second moment of the intensities of an acentric reflection for a nontwinned crystal should be two, as shown by the solid gray line. At high resolutions, where the uncertainty in the intensity becomes comparable to the mean intensity, the second moment should increase from two. This deviation is calculated from the estimated uncertainties and is shown as dotted lines for the Ev11 (blue) and MM24 (pink) protocols. The solid colored lines show the observed second moment calculated from the intensities of the reflection.

Fig. 4 ▸ shows the weighted second moment of intensities versus resolution and demonstrates consistency between intensities and uncertainties. The second-moment metric quantifies the dispersion of merged, unrelated intensities within a resolution bin. For acentric reflections, the Wilson distribution implies that it should be two, as shown by a solid black line. When the reflection uncertainty becomes comparable to the average value, measurement error begins to increase the expected dispersion from two. This expected deviation, calculated only from the estimated uncertainty after merging, is plotted for Ev11 and MM24 as blue and pink dotted lines, respectively. The observed second moments, calculated only from the intensities after merging, are plotted as solid lines. The expected and observed weighted second moments are calculated in *phenix.phaser*. Before their calculation, *phenix.phaser* performs outlier rejection and a correction for anisotropic *B* factors.

The weighted second-moment plots were a sensitive diagnostic tool for the identification of subtle artifacts in our data processing. Initially, the weighted second-moment plot for the cytochrome data set decreases from two at high resolution. This was due to a systematic underestimation of the background during integration. Reprocessing with a more appropriate integration model brought the observed and expected weighted second moments into agreement. This case demonstrates the utility of weighted second-moment plots as sensitive diagnostic tools. Its usage in macromolecular crystallography should be further explored and included in standard data-reduction software.

For cytochrome, IPNS, MMOH and sMMOox, the MM24 model provides an *I*/σ that is more consistent with CC_1/2_ and uncertainties that accurately predict the observed second moment. For thermolysin, neither model is able to produce uncertainties that are consistent with intensities (Fig. 4 ▸*a*) and *I*/σ remains large enough through the useful resolution range that it cannot be compared with CC_1/2_ (Fig. 3 ▸*f*). This data set was collected during an early era of XFEL experimentation and was recorded with a CSPAD detector. The XFEL pulse length is of the order of 10 fs, implying an enormous count rate. Developing a detector that could record XFEL pulses was an incredible technical advancement. However, the CSPAD operated in a mix-gain mode that was notoriously difficult to calibrate. We suspect, but cannot prove, that the discrepancies with thermolysin originate in issues with the detector. Fig. 3 ▸(*j*) and 4 ▸(*e*) both indicate some degree of overestimation of σ for sMMOox, but the MM24 model does make for a significant improvement over the Ev11 model.

**Figure 5:**
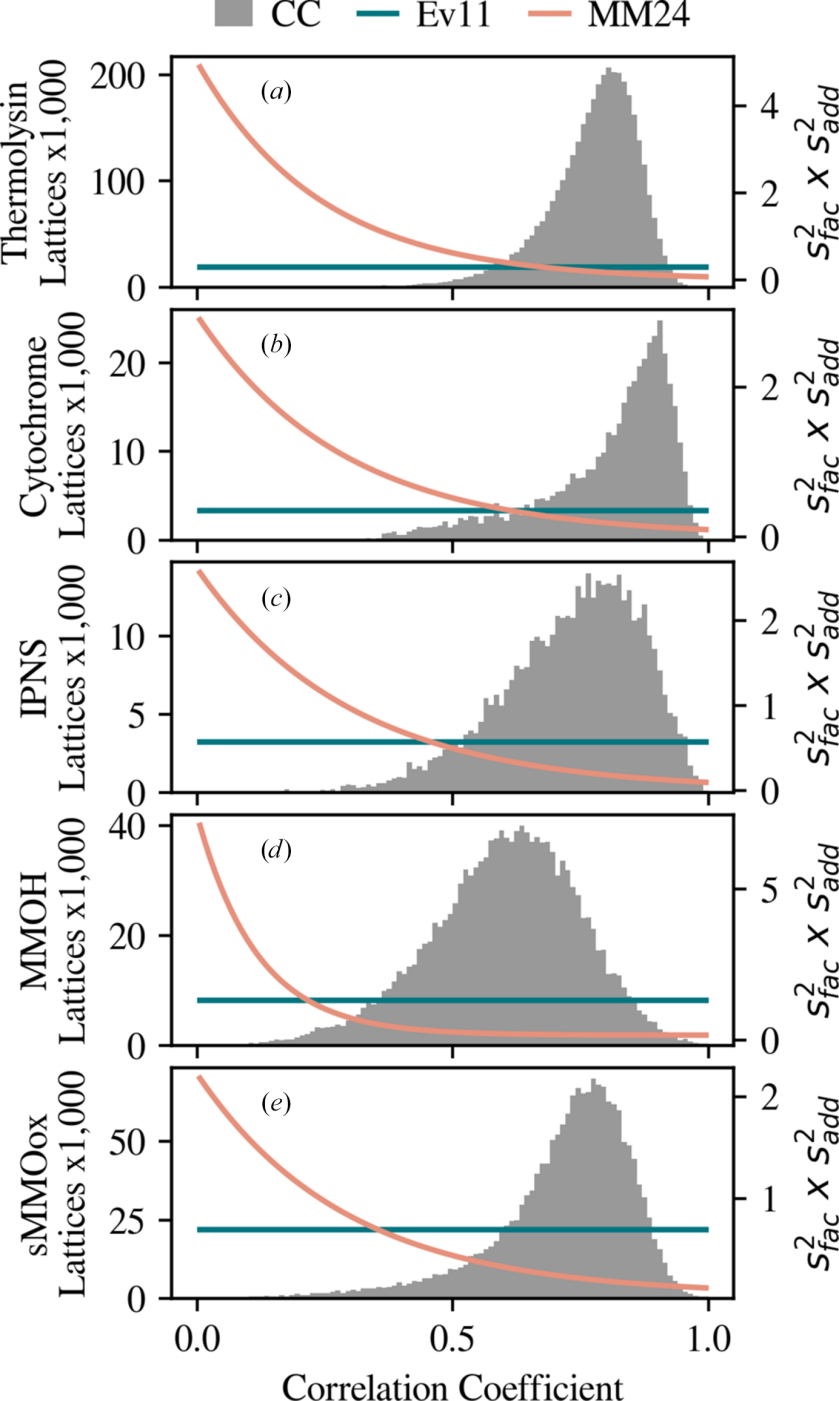
Histograms of the per-lattice correlation coefficient, CC_*c*_, for (*a*) thermolysin, (*b*) cytochrome, (*c*) IPNS, (*d*) MMOH and (*e*) sMMOox. The error term, 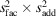, is plotted as optimized with the Ev11 and MM24 protocols in blue and pink, respectively. Relative to the Ev11 protocol, the MM24 error model has a higher adjusted error at low correlation.

Fig. 5 ▸ shows histograms of the correlation coefficient between the integrated intensities and a scaling reference, determined for each lattice in the data set. For each data set in this study, this correlation coefficient is determined after reflection intensities are scaled to a common reference and corrected for Wilson *B* factor and partiality. However, partiality correction is not always performed. For those cases, a correlation coefficient calculated after scaling can be used instead. The term 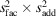 for MM24 is plotted as a function of the correlation coefficient, and compared with the constant value for Ev11, to demonstrate the degree to which low-correlation lattices are down-weighted by this parameterization. These terms were determined with the *t*-distribution likelihood with ν allowed to optimize.

These results demonstrate that the improved statistical approach of the MM24 protocol provides a more accurate calibration of measurement error than existing error models. The normalized pairwise differences as an optimization target places the focus on the scale of the data without the need for a mean estimate. The maximum-likelihood optimization utilizing a *t*-distribution provides flexibility to manage data distributed with heavier tails than a normal distribution. For all proteins except thermolysin, anomalous map heights at heavy-atom positions increased significantly. *R*_work_ and *R*_free_ showed significant improvements for cytochrome.

## 4. Discussion

This paper demonstrates the MM24 approach to error calibration for the merging of serial crystallographic data. It is distinguished from the Ev11 error model in two key ways. Firstly, it acknowledges that not all lattices are measured with the same accuracy and should not be weighted equally when merging. Appropriate error calibration can assign merging weights that reflect this variation in accuracy. MM24 does this by creating a ‘score’, a correlation coefficient in this case, which is then used to assign varying amounts of confidence to each lattice. Secondly, it utilizes robust statistics to optimize the empirical transformation of counting-statistics error to final uncertainty estimates. This is performed through a reformulation of the optimization of the error model that makes fewer and less stringent assumptions about incoming data.

The MM24 algorithm was applied to five data sets. In four cases, cytochrome, IPNS, MMOH, and sMMOox, the MM24 algorithm produced uncertainties such that the agreement between CC_1/2_ and *I*/σ and between *I* and σ improved compared with Ev11. Additionally, the comparisons between ω_*hjk*_ and its target distribution in Fig. 2 ▸ show that MM24 acts in a consistent manner. This implies accurate and consistently determined *I*/σ.

The MM24 algorithm generates *I*/σ values that are roughly 1.5 times larger than those from the Ev11 algorithm for each data set. If using *I*/σ = 2 to determine the resolution cutoff of the data set, the cytochrome data set would be cut at 2.05 and 1.75 Å for the Ev11 and MM24 algorithms, respectively. While this paper does not address appropriate uses of σ and *I*/σ values after merging, it clearly demonstrates that careful consideration of the uncertainty estimates must be performed before their downstream use.

## 5. Software availability

Instructions for downloading and using *cctbx.xfel* are available from the *cctbx.xfel* wiki at https://cci.lbl.gov/xfel. See also Brewster, Young *et al.* (2019 ▸) for instructions on using the *cctbx.xfel* graphical user interface. Documentation for *cctbx.xfel.merge* is available at https://github.com/cctbx/cctbx_project/tree/master/xfel/merging.

## Figures and Tables

**Table 1:** Integrated intensities were merged with both error models and merging results were compared through phasing with thermolysin and refinement for cytochrome, IPNS, MMOH and sMMOox Generally, the MM24 error model results in larger anomalous map height, *I*/σ and CC_1/2_.

	Thermolysin		Cytochrome		IPNS		MMOH		sMMOox	
Facility, beamline	LCLS, CXI		SWISSFEL, Bernina		LCLS, MFX		LCLS, MFX		LCLS, MFX	
Detector	CSPAD		JUNGFRAU 16M		Rayonix MX340-XFEL		Rayonix MX340-XFEL		Rayonix MX340-XFEL	
Wavelength (Å)	1.27		1.31		1.30		1.30		1.74	
cxi.db accession No.	81		229		230		230		—	
PDB code	4ow3		—		6zae		6yd0		—	
No. of lattices	164639		8631		10365		28846		38325	
No. of reflections										
Total	40619913		2243336		1984570		16079131		17459589	
Unique	101051		92647		69019		281110		225557	
Resolution (Å)	34.35–1.80 (1.83–1.80)		54.00–1.75 (1.78–1.75)		21.80–1.70 (1.73–1.70)		34.54–1.80 (1.88–1.85)		37.90–2.00 (2.04–2.00)	
Correlation filter threshold	0.1		0.3		0.1		0.1		−1	
Error model	Ev11	MM24	Ev11	MM24	Ev11	MM24	Ev11	MM24	Ev11	MM24
ν (degrees of freedom)	N/A	4.4	N/A	7.6	N/A	7.9	N/A	5.3	N/A	9.4
*I*/σ	21.94 (5.21)	32.01 (9.44)	3.019 (0.73)	5.66 (1.82)	3.26 (0.69)	4.92 (1.03)	3.14 (0.41)	6.40 (0.80)	3.62 (0.24)	5.54 (0.32)
CC_1/2_ (%)	99.9 (86.3)	99.9 (86.5)	97.5 (32.0)	98.7 (14.7)	97.8 (31.2)	98.4 (32.0)	98.1 (22.5)	98.9 (13.3)	86.4 (0.04)	99.3 (0.03)
Wilson *B* (Å^2^)	24.8	23.9	21.9	22.1	18.9	19.1	28.8	29.6	39.3	39.7
Map height (σ)	Zn	Zn	Fe	Fe	Fe	Fe	Fe	Fe	Fe	Fe
	80.1 ± 0.9	74.2 ± 3.0	15.8 ± 0.1	22.1 ± 0.1	19.1 ± 0.1	20.0 ± 0.1	17.4 ± 0.2	18.6 ± 0.3	12.3 ± 0.1	12.3 ± 0.1
							12.0 ± 0.2	13.7 ± 0.1	11.1 ± 0.1	11.7 ± 0.1
Residues built (of 316)	302 ± 6	298 ± 10	N/A	N/A	N/A	N/A	N/A	N/A	N/A	N/A
*R*_work_ (%)	19.7 ± 1.2	21.0 ± 1.7	18.0 ± 0.1	17.1 ± 0.1	15.3 ± 0.1	15.1 ± 0.1	17.8 ± 0.1	17.7 ± 0.3	18.1 ± 0.1	18.1 ± 0.1
*R*_free_ (%)	22.3 ± 1.2	23.7 ± 1.7	20.3 ± 0.4	19.2 ± 0.4	18.1 ± 0.3	17.7 ± 0.3	20.2 ± 0.4	19.8 ± 0.5	20.7 ± 0.6	20.6 ± 0.4

## Footnotes

1 *cctbx.xfel.merge* re-implements the older *cxi.merge* program described by Hattne *et al.* (2014 ▸), adding multiprocessing, and will be described more fully in an upcoming publication. See https://github.com/cctbx/cctbx_project/tree/master/xfel/merging.
